# Hachimijiogan, a traditional herbal medicine, modulates adipose cell function and ameliorates diet-induced obesity and insulin resistance in mice

**DOI:** 10.3389/fphar.2023.1167934

**Published:** 2023-05-12

**Authors:** Syota Kagawa, Katsuya Tanabe, Makoto Hiromura, Kakuyou Ogawa, Takayuki Koga, Takahiro Maeda, Kikuko Amo-Shiinoki, Hiroyuki Ochi, Yui Ichiki, Shogo Fukuyama, Saori Suzuki, Natsuki Suizu, Takaaki Ohmine, Sakurako Hamachi, Hiroshi Tsuneki, Shigeru Okuya, Toshiyasu Sasaoka, Yukio Tanizawa, Fumihiro Nagashima

**Affiliations:** ^1^ Department of Natural Products Chemistry, Daiichi University of Pharmacy, Fukuoka, Japan; ^2^ Division of Endocrinology, Metabolism, Hematological Sciences and Therapeutics, Yamaguchi University Graduate School of Medicine, Yamaguchi, Japan; ^3^ Department of Pharmaceutics and Biochemistry, Daiichi University of Pharmacy, Fukuoka, Japan; ^4^ Department of Natural Medicine, Daiichi University of Pharmacy, Fukuoka, Japan; ^5^ Department of Hygienic Chemistry, Daiichi University of Pharmacy, Fukuoka, Japan; ^6^ Department of Clinical Pharmacology, University of Toyama, Toyama, Japan; ^7^ Health Administration Centre, Organisation for University Education, Yamaguchi University, Yamaguchi, Japan

**Keywords:** traditional herbal medicine, adipocyte, leptin, obesity, insulin resistance

## Abstract

*Hachimijiogan* (HJG) has originally been used to ameliorate a variety of symptoms associated with low ambient temperatures. However, its pharmacological action in metabolic organs remains unclear. We hypothesized that HJG may modulate metabolic function and have a potential therapeutic application to metabolic diseases. To test this hypothesis, we investigated metabolic action of HJG in mice. Male *C57BL/6J* mice chronically administered with HJG showed a reduction in adipocyte size with increased transcription of beige adipocyte-related genes in subcutaneous white adipose tissue. HJG-mixed high-fat diet (HFD)-fed mice showed alleviation of HFD-induced weight gain, adipocyte hypertrophy, liver steatosis with a significant reduction in circulating leptin and Fibroblast growth factor 21 despite no changes in food intake or oxygen consumption. Feeding an HJG-mixed HFD following 4-weeks of HFD feeding, while a limited effect on body weight, improved insulin sensitivity with a reversal of decreased circulating adiponectin. In addition, HJG improved insulin sensitivity in the leptin-deficient mice without significant effects on body weight. Treatment with *n*-butanol soluble extracts of HJG potentiated transcription of Uncoupling protein 1 mediated by β3-adrenergic agonism in 3T3L1 adipocytes. These findings provide evidence that HJG modulates adipocyte function and may exert preventive or therapeutic effects against obesity and insulin resistance.

## 1 Introduction

The number of people affected by obesity worldwide has been increasing and is expected to continue (Wang et al., 2008; GBD, 2015 Obesity Collaborators, 2017). Obesity can lead to atherosclerosis and metabolic diseases due to a condition known as metabolic syndromes characterized by visceral fat accumulation and insulin resistance (Després and Lemieux, 2006). Currently, there is no cure or remission-inducing treatment for such lifestyle-related diseases. Therefore, the development of alternative treatments is urgently needed to combat obesity and metabolic diseases (Bessesen and Van Gaal, 2018).

In general, conventional western medicines have a scientific basis for their medicinal efficacy and show rapid and robust pharmacological action with respect to a single disease. Meanwhile, traditional herbal medicines are mixtures of natural herbal ingredients or processed products, and they are used for a variety of medicinal purposes based on long-standing clinical experience, owing to less adverse effect. They have advantages in treating diseases of unknown cause and for conditions in which conventional medicines are not effective. As examples of Japanese Kampo medicines that are traditional herbal medicines originally developed in Japan, *Rikkunshito* reportedly suppresses cisplatin-induced anorexia and *Goshajinkigan* ameliorates diabetic neuropathy (Nishizawa et al., 1995; Takeda et al., 2008). Given various clinical benefits, therapeutic applications of Japanese Kampo medicines are being developed. However, little is known about pharmacological mechanisms underlying the clinical effects (Ekstein and Schachter, 2010; Bi et al., 2014).

*Hachimijiogan* (HJG), one of the Japanese Kampo medicines, is composed of eight crude drugs (*Rehmanniae Radix*, *Corni Fructus*, *Dioscoreae Rhizoma*, *Alismatis Tuber*, *Poria*, *Moutan Cortex*, *Cinnamomi Cortex* and *Aconiti Radix Processa et Pulverata*). In Japan, the clinical use of HJG has been approved to alleviate various symptoms such as pollakiuria, cold sensation, and numbness, which are induced under a condition of low ambient temperature (Zhongjin, 1987; Tsumura, 1991; Sato, 2005). However, the pharmacological mechanisms underlying the observed clinical effects remain elusive. In rats, HJG administration increased the skin temperature and mitigated the detrusor overactivity caused by acute cold exposure (Imamura et al., 2013). In addition, HJG reportedly improved hyperglycemia in rats with streptozotocin-induced diabetes (Hirotani et al., 2007). Owing to the clinical effects and the experimental evidences, we have hypothesized that HJG may modulate metabolic function and have a potential therapeutic application to metabolic diseases. To test the hypothesis, in this study, we investigated the metabolic action of HJG in mice, and its preventive effects on diet-induced obesity were examined.

## 2 Materials and methods

### 2.1 Preparation of HJG extract

The powder of the aqueous extract of HJG (HJGE) was prepared and supplied by Kotaro Pharmaceutical Co., Ltd. (Osaka, Japan). It was free of other additives. The medicinal components of each of the eight crude drugs comprising HJG are listed in Supplementary Table S1. HJGE was obtained by extracting with water 10-fold the total amount of these crude drugs at 100°C for 1 h, then filtered, vacuum concentrated, and spray-dried.

### 2.2 High-performance liquid chromatography (HPLC) analysis of HJGE

Qualitative analysis of HJGE was performed using a three-dimensional HPLC (3D-HPLC) system. The dried HJGE (1 g) was extracted with 50 mL of 50% methanol by a 15 min ultrasonication, filtered, and analyzed by HPLC (EXTREMA 4000 Model; JASCO Corporation., Tokyo, Japan) under the following conditions: 20 μL of each sample was applied to an octadecylsilyl (ODS) column (L-column2 ODS, 4.6 mm × 150 mm, 5 μm; CERI., Tokyo, Japan). The elution solvents were CH_3_CN (A) and 0.05 mol/L sodium dihydrogen phosphate (SDP) solution (B). Firstly, the column was eluted by 10% A and 90% B isocratically for 5 min, then a linear gradient of, by volume, 10% A and 90% B changing over 45 min to 30% A and 70% B. The flow rate was 1.0 mL/min, and the column temperature was 25 °C. The UV spectrum ranging from 210 to 400 nm was collected with a photodiode array (PDA) detector (MD-4015; JASCO Corporation).

### 2.3 Animal model and treatment

Five-week-old male *C57BL/6J* and 7-week-old leptin deficient mice (*ob/ob*) were purchased from Japan SLC. The mice were bred in a pathogen-free environment in a temperature-controlled room at 24 °C. The mice were maintained on a 12-h light/dark cycle with a light period from 8:00 a.m. to 8:00 p.m. The mice had free access to food and water. The main ingredients of the chow diet (CE-2) and high fat diet (HFD) are shown in Supplementary Table S2 (CLEA Japan, Inc. Tokyo, Japan). The diets mixed with 3.8% (w/w) HJGE were produced by CLEA Japan, and all diets used in this study were in a pellet form. The actual dosage of HJGE was calculated by using the following formula: 0.038× food intake (g/day)/body weight (kg). After acclimatization to the environment, 6-week-old *C57BL/6J* were randomly divided into two groups and fed a CE-2 or a CE-2 containing 3.8% (w/w) HJGE for 4 weeks. For studies with HFD, 6-week-old *C57BL/6J* fed CE-2 were randomly divided into three groups and fed a CE-2, an HFD or an HFD containing 3.8% (w/w) HJGE (HFD + HJGE). Also, mice fed an HFD for 4 weeks from 6 weeks of age were similarly divided into three groups and fed a CE-2, an HFD and an HFD + HJGE. 10-week-old *ob/ob* mice acclimated for 3 weeks to the environment were fed a CE-2 or a CE-2 containing 3.8% HJGE for 10 weeks. Body weights were measured weekly throughout all animal studies. We measured the rectal temperatures at the light and dark periods using the environmental logger (AD-1687; A&D Company, Tokyo, Japan) and the probe (AX-KO4746-100; A&D Company). At the end of the experiment, all mice were euthanized with isoflurane to collect tissues and blood samples. For acute cold exposure, *C57BL/6J* fed a CE-2 or a CE-2 mixed with HJGE for 4 weeks were acutely transferred to the cold circumstance at 4°C from 24°C of ambient temperature. The mice were given free access to food and water during the cold exposure. All animal experiments were carried out with approval from the Animal Experiment Committee of Daiichi University of Pharmacy (approval number 17001).

### 2.4 Indirect calorimetry

After feeding mice the HFD or HJGE-mixed HFD for 4 weeks, they were separated and housed in individual metabolic cages to measure oxygen consumption (VO_2_), carbon dioxide output (VCO_2_), locomotor activity, and food intake (MK-5000RQ; Muromachi Kikai, Co., Ltd., Tokyo, Japan). The room temperature was maintained at 24°C throughout the experiments and measurements. HFD, HJGE-mixed HFD, and water were provided *ad libitum*. VO_2_ and VCO_2_ levels were corrected for the total body weight raised to the 0.75 power (Kon et al., 2019).

### 2.5 Measurement of blood glucose and circulating hormones

Blood samples were collected from the caudal vein of mice that were fasted for 16 h or fed randomly. Blood glucose was measured using a glucose meter (FreeStyle Freedom Lite, NIPRO, Osaka, Japan). Blood samples were centrifuged at 3,000 rpm for 10 min at 4°C to obtain serum, and fasting serum insulin level was measured using a commercial enzyme-linked immunoassay (ELISA) kit (Morinaga Institute of Biological Science, Inc., Yokohama, Japan). Homeostatic model assessment for insulin resistance (HOMA-IR) was performed using the following formula: fasting blood glucose (mg/dL) × fasting immunoreactive insulin (µU/mL)/405.

Blood samples were collected from the inferior vena cava of euthanized mice for metabolic hormone measurement, and serum samples were prepared as described earlier. Serum leptin, fibroblast growth factor 21 (FGF21), and adiponectin were measured using commercial ELISA kits (for leptin, Morinaga Institute of Biological Science, Inc.; for FGF21 and adiponectin, R&D Systems, Inc., Minneapolis, MN, United States).

### 2.6 Measurement of hepatic triglyceride content

Liver triglycerides were extracted using modified Folch’s method (Folch et al., 1957). Briefly, the mouse livers were homogenized using a solution containing chloroform and methanol at a ratio of 2:1. The homogenate was centrifuged, the chloroform layer was collected, and the chloroform was evaporated. The extracted triglycerides were redissolved in 2-propanol and measured using the LabAssay Triglyceride Kit (FUJIFILM Wako Pure Chemical Corporation, Osaka, Japan) according to the manufacturer’s instructions.

### 2.7 Fecal lipids analysis

Fresh fecal samples were dried for 24 h, and the dry weight was measured. Triglyceride was extracted from feces with 1% TritonX 100 dissolved in saline. Free fatty acids were extracted with saline. Each extract was measured using commercially available kits (triglycerides: Cell Biolabs, Inc., San Diego, CA, United States; free fatty acids: FUJIFILM Wako Pure Chemical Corporation).

### 2.8 Histological analysis and measurement of adipocyte size

The epididymal white adipose tissue (eWAT), inguinal white adipose tissue (iWAT), brown adipose tissue (BAT), and liver tissues were fixed and embedded in paraffin using 4% paraformaldehyde phosphate buffer solution. The paraffin-embedded tissue blocks were sliced and stained using a hematoxylin-eosin (HE) solution. Images of the sections were captured using a BZ-X800 microscope (Keyence Corporation, Osaka, Japan). Adipocyte diameter was measured using BZ-X800 Analyzer software (Keyence Corporation). At least 50 white adipocytes were analyzed for each mouse.

### 2.9 RNA isolation and real-time polymerase chain reaction (PCR)

The total RNA was extracted from the mouse tissues using the QIAzol reagent (Qiagen, Manchester, United Kingdom). The RNA was reverse-transcribed to cDNA using the High Capacity cDNA Reverse Transcription Kit (Thermo Fisher Scientific, Waltham, MA, United States). Real-time PCR was performed using the Light Cycler 96 System and FastStart Essential DNA Green Master (Roche Diagnostics K.K., Basel, Switzerland). Relative gene expression was calculated using the 2^−ΔΔCT^ method and normalized by the relative expression of *β-actin* for mouse tissues. For 3T3L1 adipocytes, normalization was carried out using *aP2*. The PCR program was as follows: pre-incubation at 95°C for 10 min, followed by 45 cycles of denaturation (95°C for 10 s) and annealing and extension (60°C for 30 s). The sequences of the primers used are listed in Supplementary Table S3.

### 2.10 Protein preparation and western blotting

The total protein was extracted from the mouse tissues using a radioimmunoprecipitation assay buffer (Nacalai Tesque, Inc., Kyoto, Japan). The extracted protein was separated using sodium dodecyl sulfate-polyacrylamide gel electrophoresis with a 5%–20% gradient gel and transferred onto a polyvinylidene difluoride membrane. The membrane was blocked with Blocking One (Nacalai Tesque, Inc.), followed by incubation with the corresponding primary antibodies (Supplementary Table S4) overnight at 4°C. After reacting the membrane with a secondary antibody, the target protein was detected using a chemiluminescent reagent (FUJIFILM Wako Pure Chemical Corporation). The proteins of interest were visualized using a Chemidoc imaging system (Bio-Rad Laboratories, Inc., CA, United States).

### 2.11 Insulin tolerance test (ITT)

The mice were fasted for 5 h and then intraperitoneally injected with insulin aspart (1 U/kg; Novo Nordisk A/S, Bagsvaerd, Denmark). Blood samples were collected from the caudal vein at 0, 30, 60, 90, and 120 min after injection. Blood glucose level was measured using a glucose meter (FreeStyle Freedom Lite; NIPRO).

### 2.12 Preparation of *n*-butanol soluble fraction from HJGE methanol extract

HJGE powder (100 g) was extracted with methanol (MeOH) for 1 month. After evaporation, the residue was partitioned between water and ethyl acetate (EtOAc). The water layer was then partitioned between water and *n*-butanol. Its *n*-butanol layer was evaporated to obtain 2.9 g of the crude extract. A part of the crude extract was dissolved in dimethyl sulfoxide (DMSO) and then used in the experiments. All solvents were purchased from FUJIFILM Wako Pure Chemical Corporation.

### 2.13 HPLC analysis of *n*-butanol soluble fraction from HJGE methanol extract

The HJGE or *n*-butanol soluble fraction of HJGE (1 mg) was dissolved in methanol (1 mL), filtered, and analyzed with HPLC (Prominence; SHIMADZU Corporation., Kyoto, Japan). Analytical conditions were as follows: 10 μL of each sample was applied to an ODS column (TSKgel ODS-80Ts, 4.6 mm × 250 mm, 5 μm; Tosoh Corporation., Tokyo, Japan). The elution solvents were CH_3_CN (A) and 0.05 mol/L acetic acid-ammonium acetate buffer solution (B). After isocratic elution with 10% A and 90% B for 5 min, the elution was changed in a linear gradient to 100% A over 60 min with 10% A and 90% B. The flow rate was set at 1.0 mL/min and the column temperature at 40°C. UV spectra from 200 to 400 nm were collected with a PDA detector (SPD-M20A; SHIMADZU Corporation).

### 2.14 Cell culture

Adipocyte differentiation from 3T3L1 was conducted using the standard method with 1 μM dexamethasone, 0.5 mM isobutylmethylxanthine, and 1 μg/mL insulin for 2 days. The medium was then switched to 10% fetal bovine serum (FBS)-Dulbecco’s modified Eagle medium (DMEM) containing 10 μg/mL insulin, and the cells were cultured for another 2 days (Amato et al., 2012). The differentiated adipocytes were cultured in 10% FBS-DMEM containing 10 μg/mL HJG extract or the same volume of DMSO as a vehicle, followed by 16 h of starvation. Thereafter, the cells were treated with 10 μM CL316243, a β3-adrenergic receptor agonist, or vehicle for 4 h and harvested (Mowers et al., 2013). All reagents were purchased from FUJIFILM Wako Pure Chemical Corporation.

### 2.15 Statistical analysis

Quantitative data are presented as mean ± standard deviation (SD). Significant differences were evaluated using the one-way analysis of variance (ANOVA), followed by Tukey’s *post hoc* analysis for multiple comparisons. A two-way repeated measures ANOVA was performed for comparisons between groups and among three groups, followed by Bonferroni’s and Tukey’s *post hoc* analysis for multiple comparisons, respectively. Data obtained from two comparison groups were evaluated using the two-tailed unpaired Student’s t-test. Results with *p* < 0.05 were considered significant. All statistical analyses were performed using GraphPad Prism 9 software.

## 3 Results

### 3.1 HPLC fingerprints profile of HJGE

**FIGURE 1:**
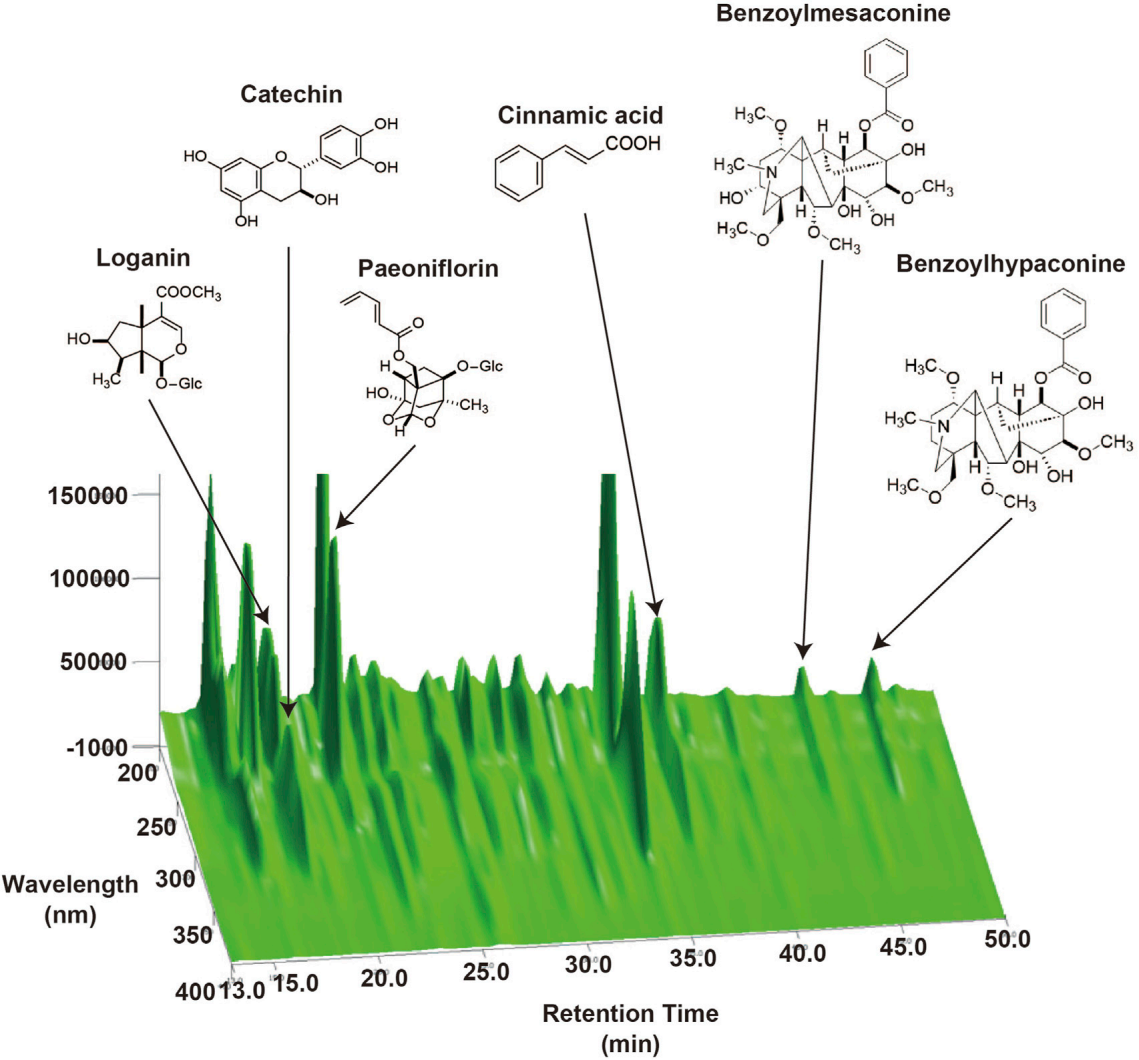
Three-dimensional high-performance liquid chromatography (3D-HPLC) profiles of HJGE.

HJG is a mixture of eight crude drugs of *Rehmanniae Radix, Corni Fructus, Dioscoreae Rhizoma, Alismatis Tuber, Poria, Moutan Cortex, Cinnamomi Coructs and Aconiti Radix Processa et Pulverata* at an indicated ratio of each (Supplementary Table S1). As shown in Figure 1, six marker compounds (loganin, catechin, paeoniflorin, cinnamic acid, benzoylmesaconine, and benzoylhypaconine) were detected from HJGE in the 3D-HPLC system with our analysis conditions. Retention times for each compound were 16.3, 17.9, 20.6, 36.4, 39.9, and 46.8 min.

### 3.2 HJG alters cell size and metabolic gene expression in WAT

**FIGURE 2:**
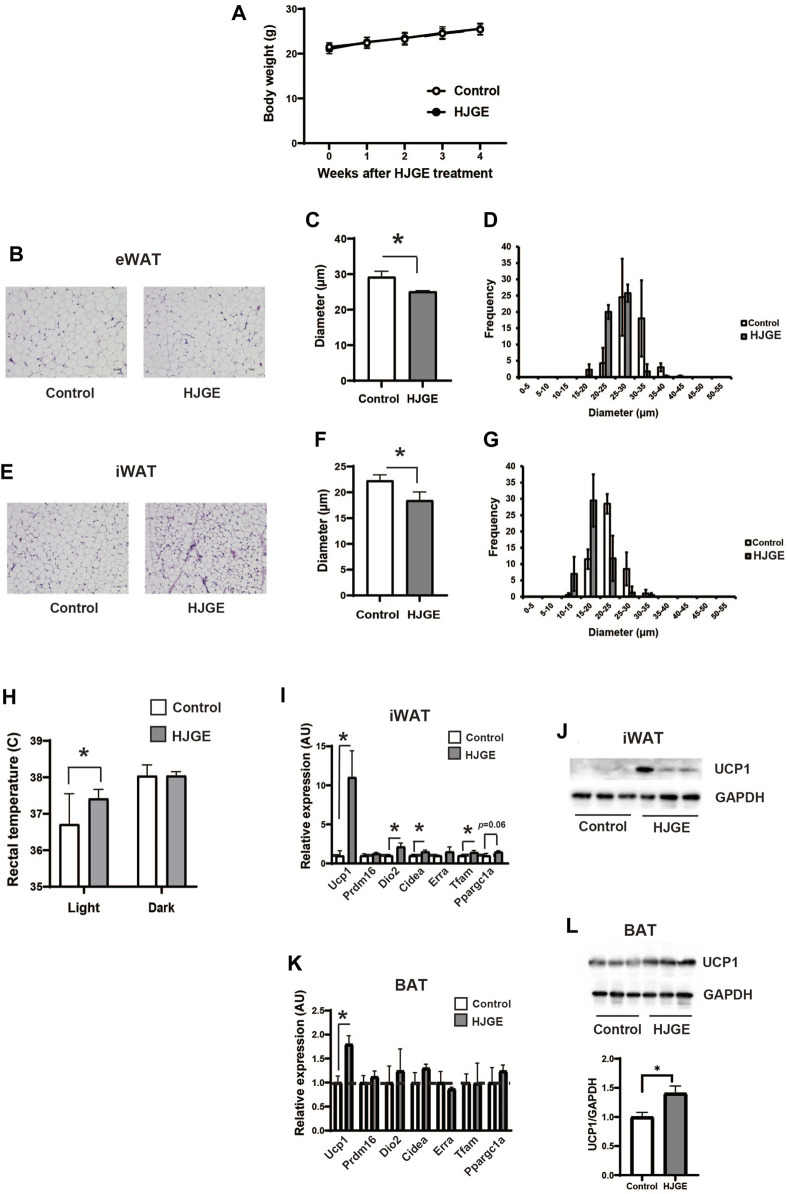
HJGE reduces the size of adipocytes and increases UCP1 expression in the iWAT of *C57BL/6J* mice. Six-week-old male *C57BL/6J* mice were fed a chow diet (control) or chow supplemented with 3.8% HJGE (HJGE) for 4 weeks. **(A)** Change in body weight (*n* = 9). **(B)** Representative hematoxylin and eosin (HE)-staining images of the eWAT. Scale bar, 100 μm. **(C)** Mean cell diameter and **(D)** cell diameter distribution in the eWAT (*n* = 4). **(E)** Representative HE-staining images of the iWAT. Scale bar, 100 μm. **(F)** Mean cell diameter and **(G)** cell diameter distribution in the iWAT (*n* = 4). **(H)** Rectal temperature (*n* = 7–8) of *C57BL/6J* mice was measured in the light and dark phases. **(I)** mRNA level of beige adipocyte-related genes in the iWAT (*n* = 3). **(J)** Protein level of UCP1 in the iWAT (*n* = 3). **(K)** mRNA levels of thermogenic genes in the BAT (*n* = 3). (**L**) Protein levels of UCP1 in the BAT (*n* = 3). Data are shown as mean ± SD. *p < 0.05; two-way ANOVA followed by Bonferroni’s *post hoc* test **(A)**; two-tailed unpaired Student’s *t*-test (**C,F,H,I,K,L**).

We first examined the effects of chronic administration of 3.8% HJGE mixed with a chow diet in mice. The actual dosage of HJGE was 4.98 ± 0.51 (mean value ± SD, *n* = 6) g/kg/day. HJGE did not alter body weight or food intake (Figure 2A; Supplementary Figure S1). However, HJGE-diet supplementation decreased the diameter of adipocytes in the eWAT (Figures 2B–D) and iWAT (Figures 2E–G) compared with chow diet feeding. Interestingly, the rectal temperature during the light cycle was increased by HJGE supplementation (Figure 2H). To assess the effects of HJGE on WAT metabolic properties, gene expression in the iWAT was examined. The expression of genes related to beige adipocytes, such as Uncoupling protein 1 (*Ucp1*), type II iodothyronine deiodinase (*Dio2*), cell death-inducing DFFA-like effector A (*Cidea*), and mitochondrial transcription factor A (*Tfam*), significantly increased in the HJGE group compared with that in the chow diet group (Figure 2I). In addition, UCP1 protein expression was detectable in the iWAT of mice fed HJGE (Figure 2J). For effects of HJGE in BAT, only *Ucp1* transcription among thermogenic genes tested was significantly increased along with an increase in its protein expression (Figures 2K, L). These results suggested that HJG modulates the properties of beige adipocytes in WAT and the thermogenic function of BAT.

### 3.3 HJG prevents obesity in mice fed an HFD

**FIGURE 3:**
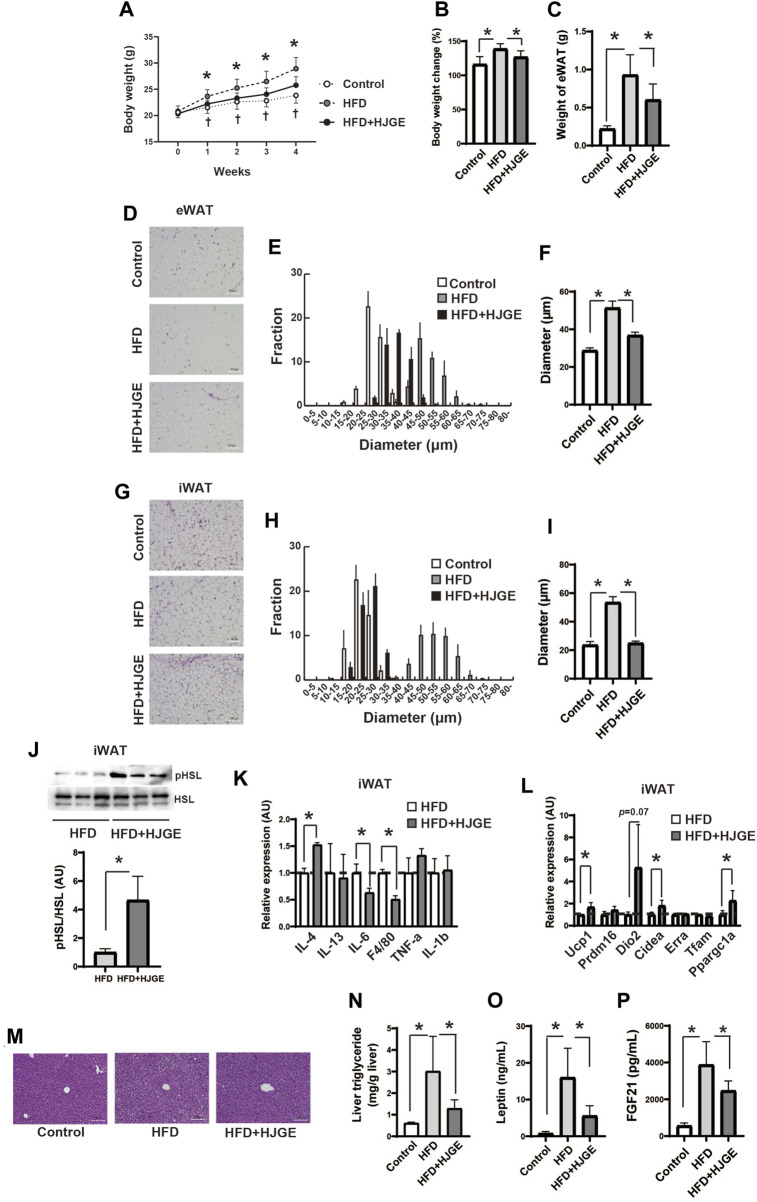
HJGE prevents HFD-induced obesity.Effects of HFD feeding on male *C57BL/6J* mice administered 3.8% HJGE versus those not administered HJGE. **(A)** Change in body weight during the observation period and **(B)** relative change in body weight from baseline to the end of the observation period (*n* = 7–9). **(C)** eWAT mass following 4 weeks of HFD feeding (*n* = 5–9). **(D)** Representative HE-staining images of eWAT. Scale bar, 100 μm. **(E)** Cell diameter distribution in eWAT and **(F)** the mean cell diameter (*n* = 4). **(G)** Representative HE-staining images of iWAT. Scale bar, 100 μm. **(H)** Cell diameter distribution in iWAT and **(I)** the mean cell diameter (*n* = 4). **(J)** Representative immunoblots of HSL phosphorylation (Ser660) in iWAT and quantification of the relative abundance (*n* = 3). **(K)** mRNA levels of anti- and pro-inflammatory markers in iWAT (*n* = 3). (**L**) mRNA levels of beige adipocyte-related genes in iWAT (*n* = 3, 4). (**M**) Representative HE-staining images of the liver. Scale bar, 100 μm. (**N**) Triglyceride level in the liver (*n* = 5–8). (**O**) Serum leptin concentration (*n* = 4–5). Graphical data are expressed as mean ± SD. *p < 0.05; two-way ANOVA followed by Tukey’s *post hoc* test (**A**; ^**†**^ *p* < 0.05 control vs. HFD), one-way ANOVA followed by Tukey’s *post hoc* test (**B,C,F,I,N,O,P**), two-tailed unpaired Student’s *t*-test (**J,K,L**).

We next examined the metabolic effects of HJG in HFD-fed *C57BL/6J* mice. Weight gain owing to HFD feeding was significantly suppressed by supplementation of 3.8% HJGE, with no associated alteration in food intake or locomotor activity (Figures 3A, B, Supplementary Figures S2A, B). The HJGE dosage via the mixed HFD was 1.86 ± 0.38 (mean ± SD, *n* = 7) g/kg/day. HJGE inhibited the HFD-induced increase in eWAT mass and ameliorated adipocyte hypertrophy with enlarged unilocular lipid droplets (Figures 3C–F). The same effects were also demonstrated in the iWAT (Figures 3G–I). Prevention of WAT hypertrophy was associated with a significant increase in the phosphorylation of Ser660 on hormone-sensitive lipase (HSL), which activates HSL enzymatic activity, and with alleviation of local inflammation assessed by changes in gene expression of the pro- and anti-inflammation markers (Figures 3J, K). Furthermore, iWAT of mice fed a mixed diet exhibited increased transcription of *Ucp1*, *Cidea* and peroxisome proliferator-activated receptor gamma coactivator 1-alpha (*Ppargc1a*) compared with mice fed an HFD. (Figure 3L). In addition to the effects on iWAT, HJGE alleviated liver steatosis (Figures 3M, N). To assess endocrine function of iWAT and liver in response to metabolic demand, plasma leptin (Frederich et al., 1995) and FGF21 derived from liver (Flippo and Potthoff, 2021) were measured, respectively. HJGE resolved the marked increase in circulating leptin and FGF21 caused by the HFD (Figures 3O, P). We then examined the potential pharmacological mechanisms whereby HJGE prevents HFD-induced obesity. However, whole metabolic respiration was not altered by HJGE (Supplementary Figures S2C, D). HJGE also did not affect lipid excretion (Supplementary Figures S2E, F).

### 3.4 HJG alleviates insulin resistance in mice with diet-induced obesity

**FIGURE 4:**
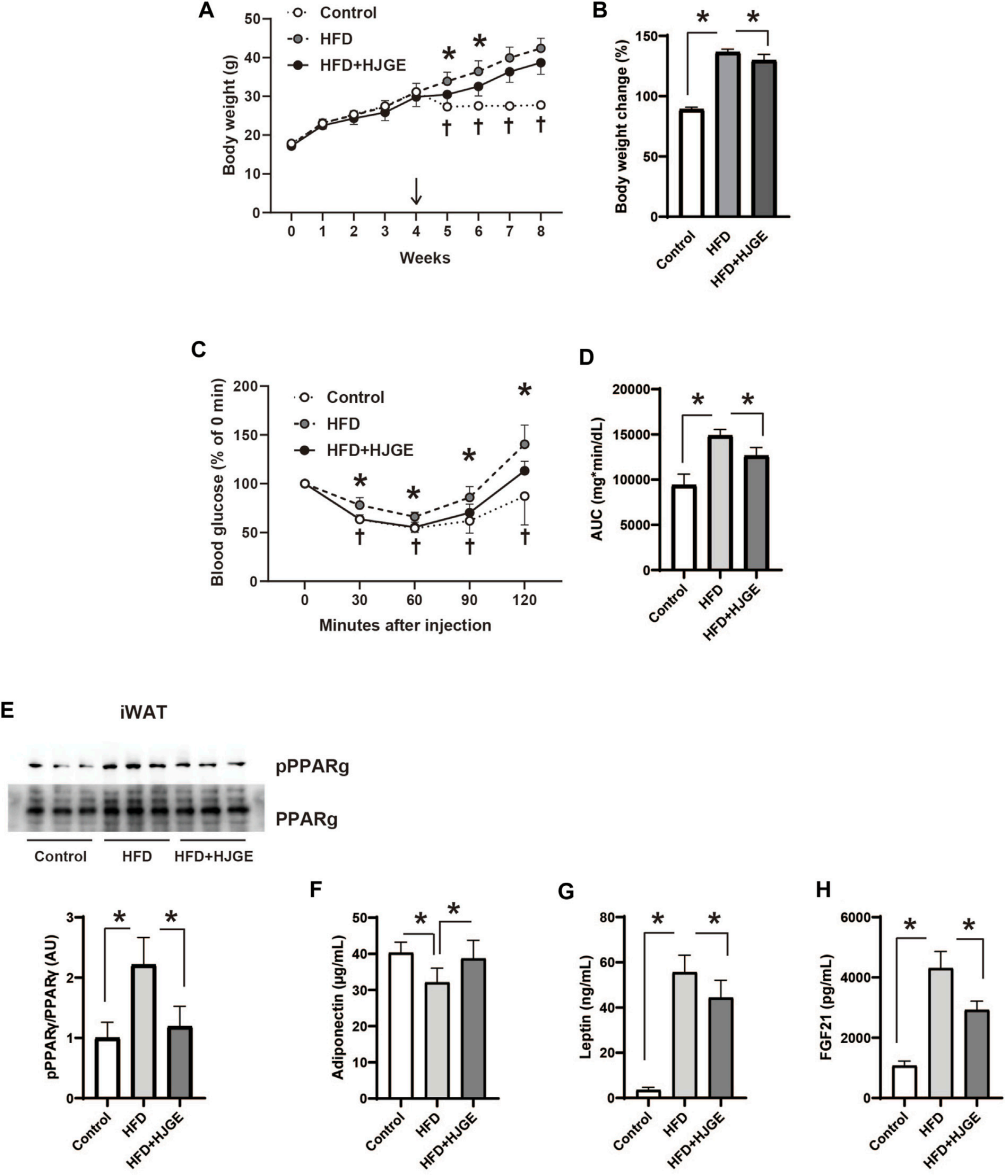
Therapeutic effects of HJGE against HFD-induced obesity. Six-week-old male *C57BL/6J* mice were fed an HFD for 4 weeks and then divided into the three diet groups administered chow diet (control), the same HFD (HFD), and HFD containing 3.8% HJGE (HFD+HJGE). **(A)** Changes in body weight during the observation period and **(B)** the relative change at the end of the observation period from body weight measured after 4 weeks of HFD feeding (*n* = 5–7). **(C)** Blood glucose level measured at the indicated time points after insulin challenge and **(D)** comparison of the area under the curve (AUC) of blood glucose level (*n* = 5–7). **(E)** Immunoblots of phosphorylated Ser273 on PPARγ in iWAT and quantification of the relative abundance (*n* = 3). The serum concentration of **(F)** adiponectin, **(G)** leptin, and **(H)** FGF21 (*n* = 4–7). Graphical data are shown as mean ± SD. *p < 0.05; two-way ANOVA followed by Tukey’s *post hoc* test (**A**, **C**; ^†^ *p* < 0.05 control vs. HFD), one-way ANOVA followed by Tukey’s *post hoc* test **(B,D–H)**.

We next examined the effects of additional HJG administration in diet-induced obese mice. *C57BL/6J* mice were fed an HFD for 4 weeks, and then divided into three diet groups, in which they were fed a chow diet (control), the same HFD, or the HFD supplemented with 3.8% HJGE (HFD+HJGE) for another 4 weeks. Weight gain relative to baseline level (start of the intervention) was suppressed by HJGE until 2 weeks, whereas the effect on body weight was reduced by the end of the observation period (Figures 4A, B). The ITT performed at the end of the observation period (4 weeks) demonstrated that the glucose-lowering action of insulin was improved in mice fed HFD + HJGE compared with that in mice fed the HFD alone (Figures 4C, D). Although the fasting serum insulin levels were not altered, HJGE significantly reduced the fasting blood glucose level with a consequent reduction in HOMA-IR (Supplementary Figures S3A–C). To gain insights into the mechanisms underlying these effects of HJGE on glucose metabolism, the phosphorylation of Ser273 on peroxisome proliferator-activated receptor γ (pPPARγ), an indicator of whole-body insulin resistance (Choi et al., 2010), was examined. As shown in Figure 4E, the pPPARγ level increased in iWAT of mice fed the HFD compared with that of control mice and was significantly reduced in mice fed HFD+HJGE. Importantly, reduced pPPARγ was associated with a reversal of the decreased circulating adiponectin level (Figure 4F). Conversely, a marked increase in circulating leptin and FGF21 levels were partially but significantly suppressed by HJGE (Figures 4G, H). Moreover, HJGE inhibited BAT hypertrophy caused by HFD feeding and alleviated the enlargement of lipid droplets in BAT adipocytes (Supplementary Figures S3D, E).

### 3.5 HJG improved insulin sensitivity in leptin-deficient mice

**FIGURE 5:**
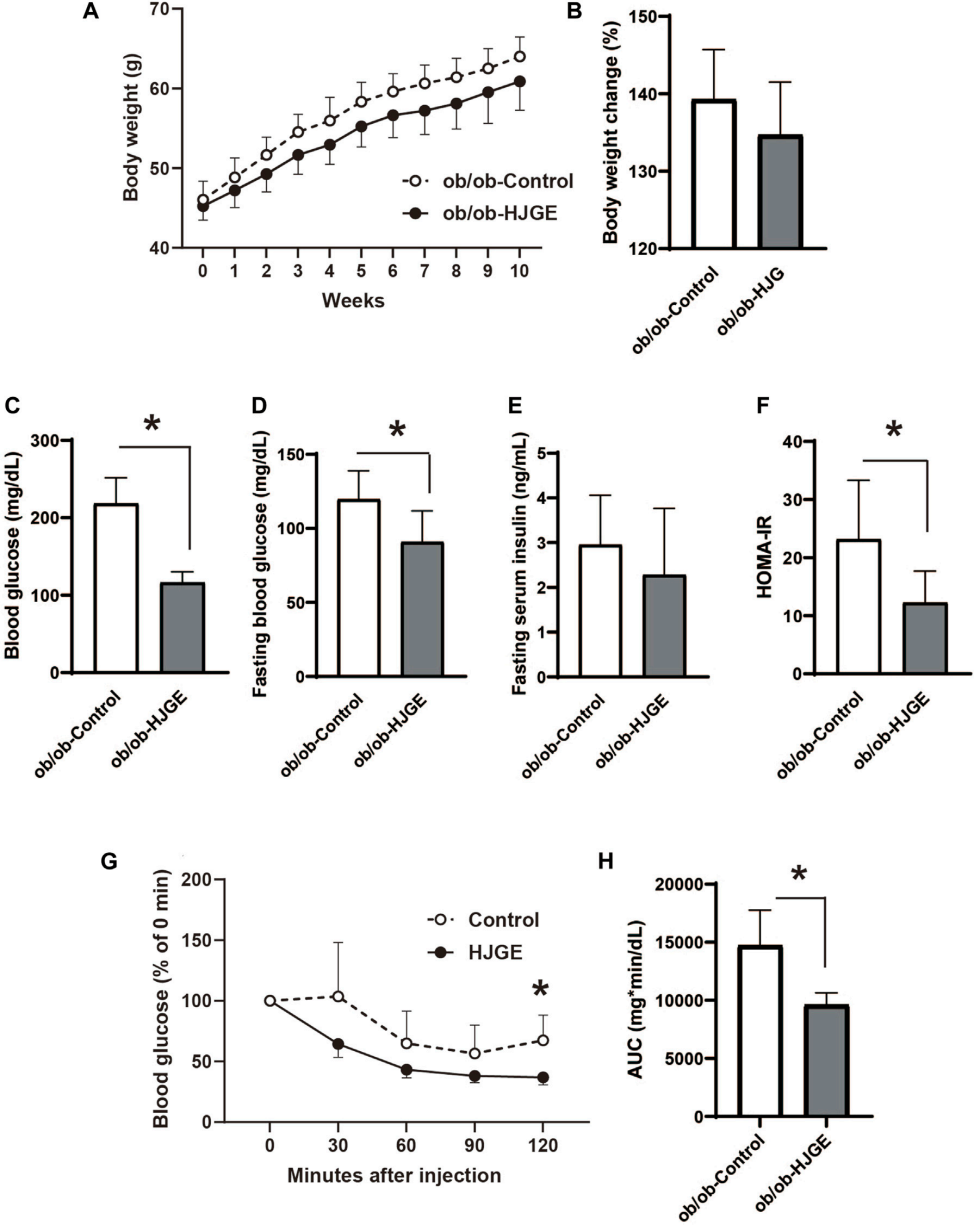
Metabolic effects of HJGE in leptin-deficient mice. 10-week-old *ob/ob* mice were fed a chow diet supplemented with or without 3.8% HJGE for 10 weeks. **(A)** Change in body weight and **(B)** relative change in body weight from the baseline (*n* = 7). **(C)** Randomly fed blood glucose, **(D)** fasting blood glucose, **(E)** fasting serum insulin level, **(F)** HOMA-IR (*n* = 7). **(G)** Change in blood glucose level measured at the indicated time points after insulin challenge and **(H)** comparison of the AUC of blood glucose level (*n* = 4–7). Graphical data are expressed as mean ± SD. *p < 0.05; two-way ANOVA followed by Bonferroni’s *post hoc* test **(A,G)**; two-tailed unpaired Student's *t*-test **(B–F,H)**.

To explore the role of leptin in the metabolic actions of HJG, 3.8% HJGE was administered to leptin-deficient mice (*ob/ob*) fed a chow diet (control). There was no significant change in body weight over the observation period (Figures 5A, B). The effects on glucose metabolism were assessed at the end of the observation period. Although there was no alteration in the fasting serum insulin level or liver triglyceride content, HJGE significantly reduced the fasting and casual blood glucose levels with a consequent reduction in HOMA-IR (Figures 5C–F; Supplementary Figure S4). Consistent with these findings, the ITT demonstrated that the blood glucose level after insulin challenge was significantly reduced in *ob/ob* mice fed HJGE compared with that in the control group (Figures 5G, H). Thus, HJGE improved whole-body insulin sensitivity in a leptin-deficient state without affecting body weight.

### 3.6 HJG directly targets adipocytes and potentiates β3-adrenergic signaling-mediated *Ucp1* induction

**FIGURE 6:**
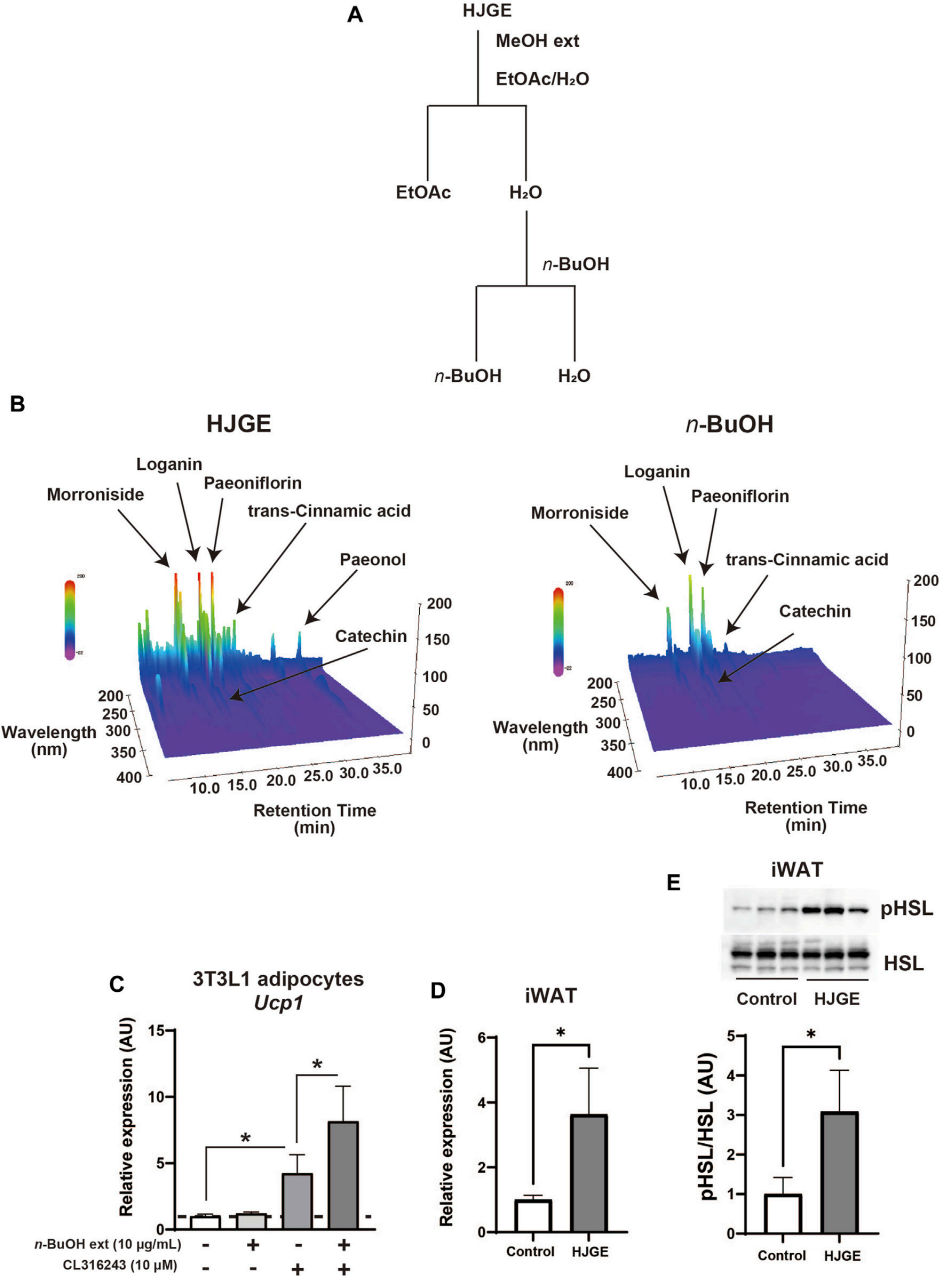
*In vitro* and *in vivo* action of HJGE onto adipocytes **(A)** Flowchart of the extraction processes of HJGE. **(B)** 3D-HPLC chart of HJGE and *n*-butanol soluble fraction of HJGE. 3T3L1 adipocytes were cultured in a medium supplemented with 10 μg/mL *n*-butanol (*n*-BuOH) soluble fraction of HJGE. At 4 h before harvesting cells, either 10 μM CL316243 or a vehicle was added to the culture medium. **(C)** *Ucp1* expression corrected by *aP2* is shown in the graph as mean ± SD (*n* = 3–5). **(D)** mRNA levels of *Ucp1* (*n* = 4), and **(E)** representative immunoblots of HSL phosphorylation (Ser660) and quantification of the relative abundance (*n* = 3) in iWAT after cold stimulation for 20 h at 4°C. One-way ANOVA followed by Tukey’s *post hoc* test **(C)**; two-tailed unpaired Student’s *t*-test **(D,E)**. *p < 0.05. MeOH, methanol; EtOAc, ethyl acetate; *n*-BuOH, *n*-butanol; aP2, adipocyte protein 2.

Finally, we examined whether HJG directly acts on adipocytes. To this end, HJGE was extracted by methanol and then separated with ethyl acetate. The aqueous layer was further separated with *n*-butanol. The organic layers dissolved in ethyl acetate or *n*-butanol were lyophilized and redissolved in DMSO (Figure 6A). A comparison of 3D-HPLC charts of HJGE and the *n*-butanol soluble fraction in the methanol extract of HJGE is shown in Figure 6B. The retention times for each compound are as follows (morroniside: 12.1 min, loganin: 16.3 min, catechin: 17.0 min, paeoniflorin: 18.6 min, trans-cinnamic acid: 22.7 min, paeonol: 35.3 min). The effects of the soluble extracts on *Ucp1* transcription were examined in 3T3L1 adipocytes. The ethyl acetate soluble fraction had no effect (data not shown). Although the *n*-butanol soluble fraction alone had no effect on *Ucp1* transcription, it synergistically potentiated *Ucp1* transcription in the presence of CL316243, a β3-adrenergic receptor agonist (Figure 6C). For *in vivo* relevance of the effect of *n*-butanol soluble fraction on *Ucp1* expression, administration with HJGE potentiated Ucp1 expression in BAT of *C57BL/6J* (Figures 2K, L), and this effect was not compromised even in the setting of leptin deficiency (Supplementary Figures S5A, B). To obtain further relevance to the regulation by sympathetic activity, we tested the effects of HJGE under acute cold stress. The mice chronically administered with HJGE exhibited enhanced *Ucp1* gene expression and phosphorylation of HSL in iWAT (Figures 6D, E). These results suggest that HJG directly targets adipocytes and likely contains some natural compounds that potentiate β3-adrenergic receptor signaling-induced *Ucp1* expression.

## 4 Discussion

HJG has been shown to be clinically effective against numerous symptoms, including frequent urination in elderly individuals, cold sensation, and numbness of the extremities. Moreover, the clinical or preclinical effects of HJG against distinct diseases, including osteoporosis, diabetic nephropathy, and dementia, have been reported (Chen et al., 2005; Nakagawa et al., 2005; Kubota et al., 2017). However, the pharmacological action accounting for the clinical effects remains unclear. In this study, we investigated the metabolic properties of HJG in lean and obese mice. First, in lean mice, chronic administration of HJGE decreased the adipocyte size in the WAT and promoted the expression of beige adipocyte signature genes in the iWAT. Second, HJGE suppressed weight gain, adipocyte hypertrophy, and liver steatosis in HFD-fed mice. Third, HJGE improved insulin resistance in HFD-induced obese mice and mice with leptin deficiency. Finally, we found that HJG contains some herbal compounds that potentiated β3-adrenergic signaling-mediated *Ucp1* transcription in differentiated adipocytes.

The results of our study showed that HJGE reduced the size of WAT and promoted the expression of beige adipocyte-related genes in subcutaneous WAT (scWAT), suggesting that the adipose tissues are an important site for HJG to exert its metabolic action. However, the identity of the components in HJG that induce these effects in adipocytes has not been fully elucidated. HJG is composed of eight different kinds of crude drugs, including *Aconitum* alkaloids at a non-toxic dose (Hikino et al., 1977; Singhuber et al., 2009) and cinnamon derivatives. For example, the administration of processed aconite root reportedly increased the body temperature of mice continuously housed under mild cold conditions (Makino et al., 2009). Moreover, the administration of cinnamon derivatives has been reported to suppress weight gain in rodents with increased *Ucp1* expression in scWAT (Jiang et al., 2017; Kwan et al., 2017). Interestingly, our observation that the *n*-butanol soluble fraction of HJGE potentiated *Ucp1* transcription induced by β3-adrenergic stimulation in differentiated adipocytes suggests that HJG may contain some chemical components that directly act on adipocytes.

The 3D-HPLC analysis revealed that HJGE and its *n*-butanol soluble fraction contained morroniside, paeoniflorin, *trans*-cinnamic acid and loganin as major natural compounds. Of these ingredients, paeoniflorin reportedly alleviated weight gain and liver steatosis in HFD fed mice (Zhang et al., 2015). In addition, it has been shown that *trans*-cinnamic acid induces brown like-phenotype in 3T3L1 white adipocytes and activated HIB1B brown adipocytes (Kang et al., 2019). But metabolic effects of morroniside and loganin are yet to be understood. Moreover, these glycosides would be metabolized in gut when orally administered. Therefore, to gain further insights of pharmacological action of HJGE, further study that focus on actions of not only the glycosides such as morroniside, loganin and paeoniflorin but also their metabolites will be required. Meanwhile, roles of other natural ingredients contained in HJGE remain unclear at this stage. With this regard, the future study that conduct comprehensive analyses of the components of HJGE could help the understanding of the underlying pharmacological mechanisms and facilitate the discovery of novel natural compounds.

HJGE prevented weight gain while inhibiting adipocyte hypertrophy when introduced concomitantly with HFD feeding. HJGE treatment also alleviated inflammation in the iWAT, liver steatosis, and elevation of circulating leptin and FGF21 levels. The prevention of adipocyte hypertrophy was associated with an increase in activating the phosphorylation of HSL (Carmen and Víctor, 2006). However, HJGE did not affect whole-body metabolic respiration, suggesting that it may suppress adipocyte hypertrophy by promoting lipolysis rather than oxidative metabolism. The phosphorylation of HSL is activated by sympathetic nerve inputs (Bartness et al., 2014), whereas HJGE may lower sympathetic tones by alleviating obesity and a paucity of leptin action. This hypothesis on HSL activation should be tested in future studies.

The effect of HJGE supplementation on body weight were limited once obesity was established following HFD feeding (Figures 4A, B). This suggests that the anti-obese effect of HJGE could be attenuated under the pathophysiological condition. Indeed, HJGE had no effects on body weight also in *ob/ob* mice, suggesting that leptin action would be necessary for the weight-reducing effects of HJGE. Owing to these observations, we speculate that decreased leptin action may attenuate metabolic action of HJGE in HFD-induced obesity. In contrast, whole body insulin sensitivity was improved by HJGE in *ob/ob* mice, suggesting a leptin-independent action. Although precise mechanisms underlying this effect remain unclear, HJGE decreased the phosphorylation of Ser273 of PPARγ in iWAT of HFD-fed mice and thereby reversed a decrease in circulating adiponectin levels. Notably, HJGE administration considerably prevented the whitening of BAT in mice with HFD-induced obesity. In line with the findings of previous studies (Choi et al., 2010; Stanford et al., 2013), these results suggest that qualitative improvement of adipocytes may at least partially account for the effects of HJGE on insulin sensitivity in diet-induced obese mice. Furthermore, the glucose-lowering effect of HJGE noted in *ob/ob* mice appeared more potent than those noted in mice with HFD-induced obesity. A substantial reduction in fasting blood glucose was observed after 30 weeks of HJGE administration (data not shown). Such sustained effects on blood glucose strongly suggest that in addition to adipocytes, other metabolic organs such as the liver, muscle, and brain could be involved in the anti-diabetic action of HJGE in leptin deficiency. Our data demonstrated that HJGE exerts leptin-dependent and -independent action in the obese mice. The latter benefits to whole body insulin sensitivity and glycemic control under the condition of decreased leptin action. With the effects, we speculate that HJG may also benefit to metabolic health in non-obese insulin resistant individuals who have a leptin paucity. Hence, future study that focus on the leptin-independent metabolic action of HJG may guide a novel therapeutic strategy for maintaining metabolic health.

The recent studies have adopted 1%–4.8% (w/w) HJG mixed diets to elucidate medicinal effects in various rodent disease models (Kubota et al., 2017; Qu et al., 2020; Ito et al., 2022). But the HJG dosages in these animal studies and our work, while no toxic effects observed, exceed the clinical dose. The rationale of the drug dosage adopted in our study is not sufficient because we have not tested the effects at different doses. Hence, the results of the present study are limited to provide adequate preclinical data, and future study to determine the optimal supplementation doses will be required to elucidate the preclinical effects of HJG.

## 5 Conclusion

Our work revealed that *Hachimijiogan*, a Japanese Kampo medicine, exerts metabolic effects in mice and prevented diet**-**induced obesity and modulated whole body insulin sensitivity. Our findings also suggested that the effect of HJG on body weight would be attenuated once obesity is established. Although the dosage of HJG used in the present study was higher than that of a clinical use, preclinical evidence obtained in the current study warrants the development of clinical application of HJG as adjuvant therapy for obesity. To this end, future study that focus on the pharmacological mechanism underlying *in vivo* effects will be needed. In addition, identifying active components in HJG may lead the development of novel therapeutics against obesity and related diseases.

## Data Availability

The original contributions presented in the study are included in the article/Supplementary Material, further inquiries can be directed to the corresponding authors.
