# Supplementary material for: Hachimijiogan, a traditional herbal medicine, modulates adipose cell function and ameliorates diet-induced obesity and insulin resistance in mice
Source: Front Pharmacol. 2023 May 12;14:1167934. doi: 10.3389/fphar.2023.1167934 (PMC10217779; doi:10.3389/fphar.2023.1167934)
Supplement: Supplementary file 1 [file DataSheet1.docx]

Supplementary Material

Hachimijiogan, a traditional herbal medicine, modulates adipose cell function and ameliorates diet-induced obesity and insulin resistance in mice

**Syota Kagawa^*^, Katsuya Tanabe^*^, Makoto Hiromura, Kakuyou Ogawa, Takayuki Koga, Takahiro Maeda, Kikuko Amo-Shiinoki, Hiroyuki Ochi, Yui Ichiki, Shogo Fukuyama, Saori Suzuki, Natsuki Suizu, Takaaki Ohmine, Sakurako Hamachi, Hiroshi Tsuneki, Shigeru Okuya, Toshiyasu Sasaoka, Yukio Tanizawa, and Fumihiro Nagashima**

*** Correspondence:**Dr. Syota Kagawa
skagawa130@gmail.com

Dr. Katsuya Tanabe
ktanabe@yamaguchi-u.ac.jp

# Supplementary Figures and Tables

## Supplementary Figures


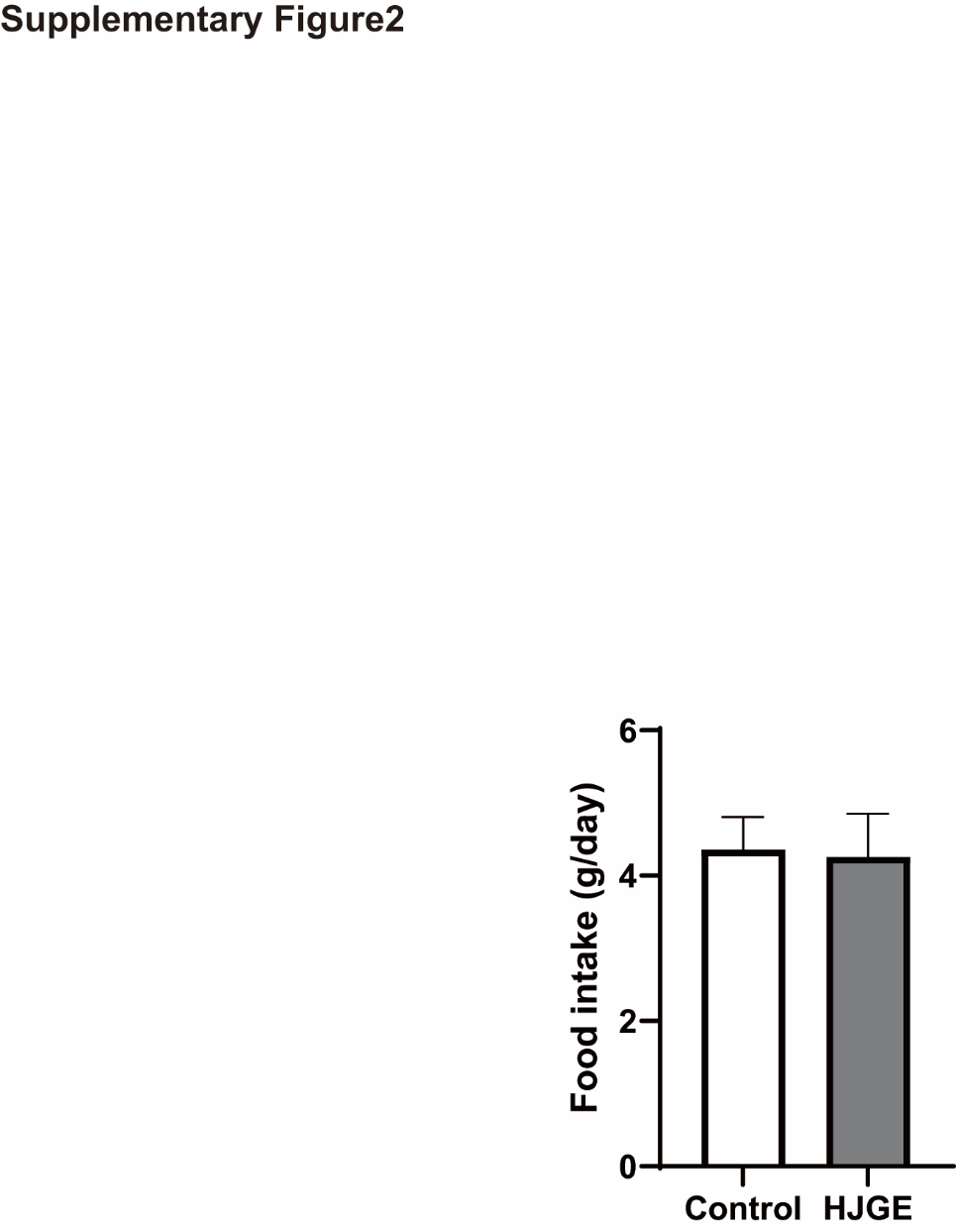


**Supplementary Figure 1.** **HJGE does not affect food intake.**

Food intake of *C57BL/6J* mice fed a chow diet (control) with or without HJGE (n = 9). Data are shown as mean ± SD. Two-tailed unpaired Student's *t-*test. **P* < 0.05.


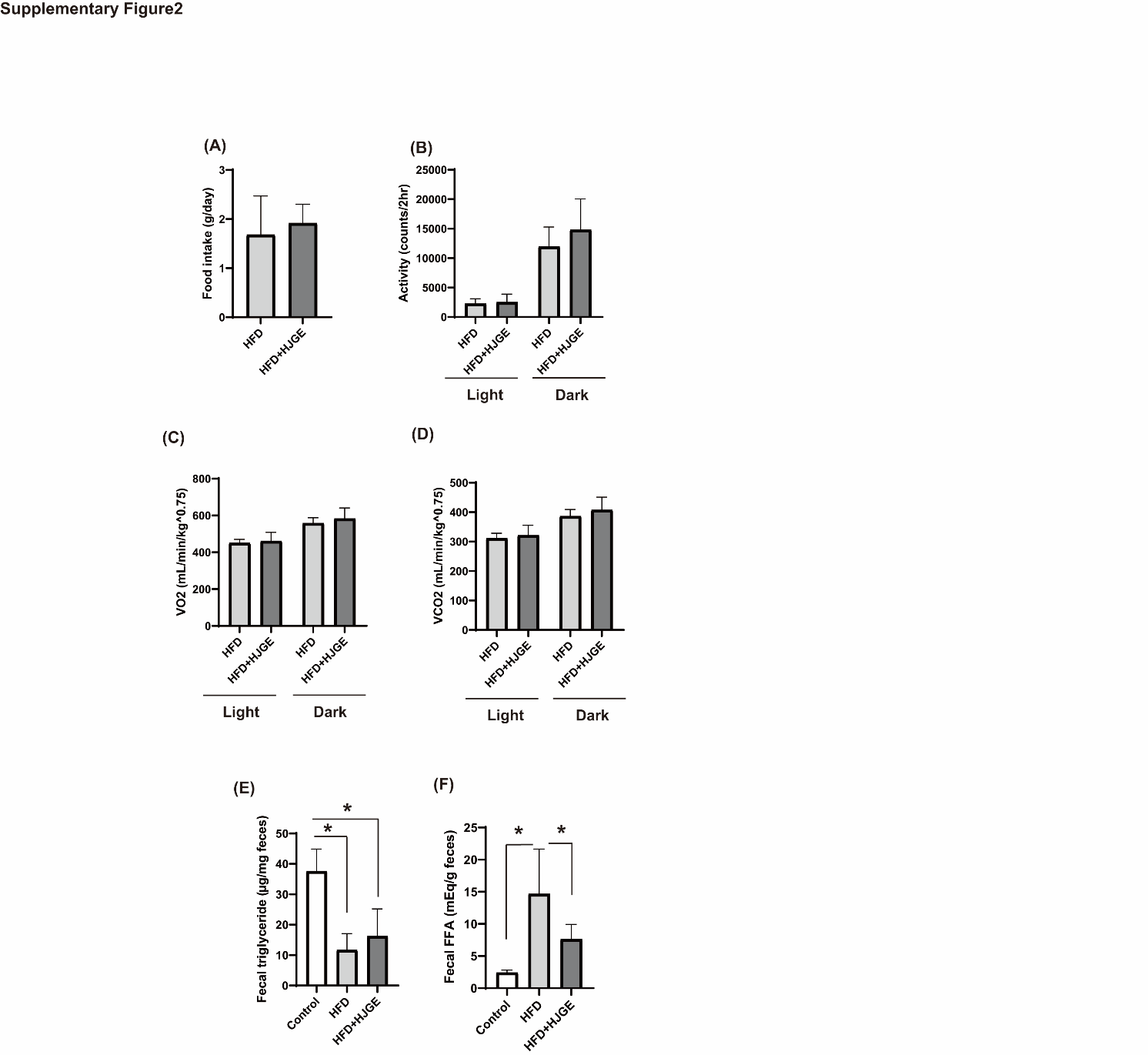
**Supplementary Figure 2.** **Effects of HJGE on food intake, activity, energy expenditure, and lipid excretion during high-fat diet feeding.**

(A) Food intake and (B) locomotor activities were measured at four weeks after HFD feeding (n = 9). (C) O_2_ consumption and (D) CO_2_ production (n = 9). (E) Triglyceride content and (F) free fatty acid content in the feces (n = 5-6). Data are shown as mean ± SD. (A, B, C, D) Two-tailed unpaired Student's *t-*test. (E, F) One-way ANOVA followed by Tukey’s post hoc test. **P* < 0.05.


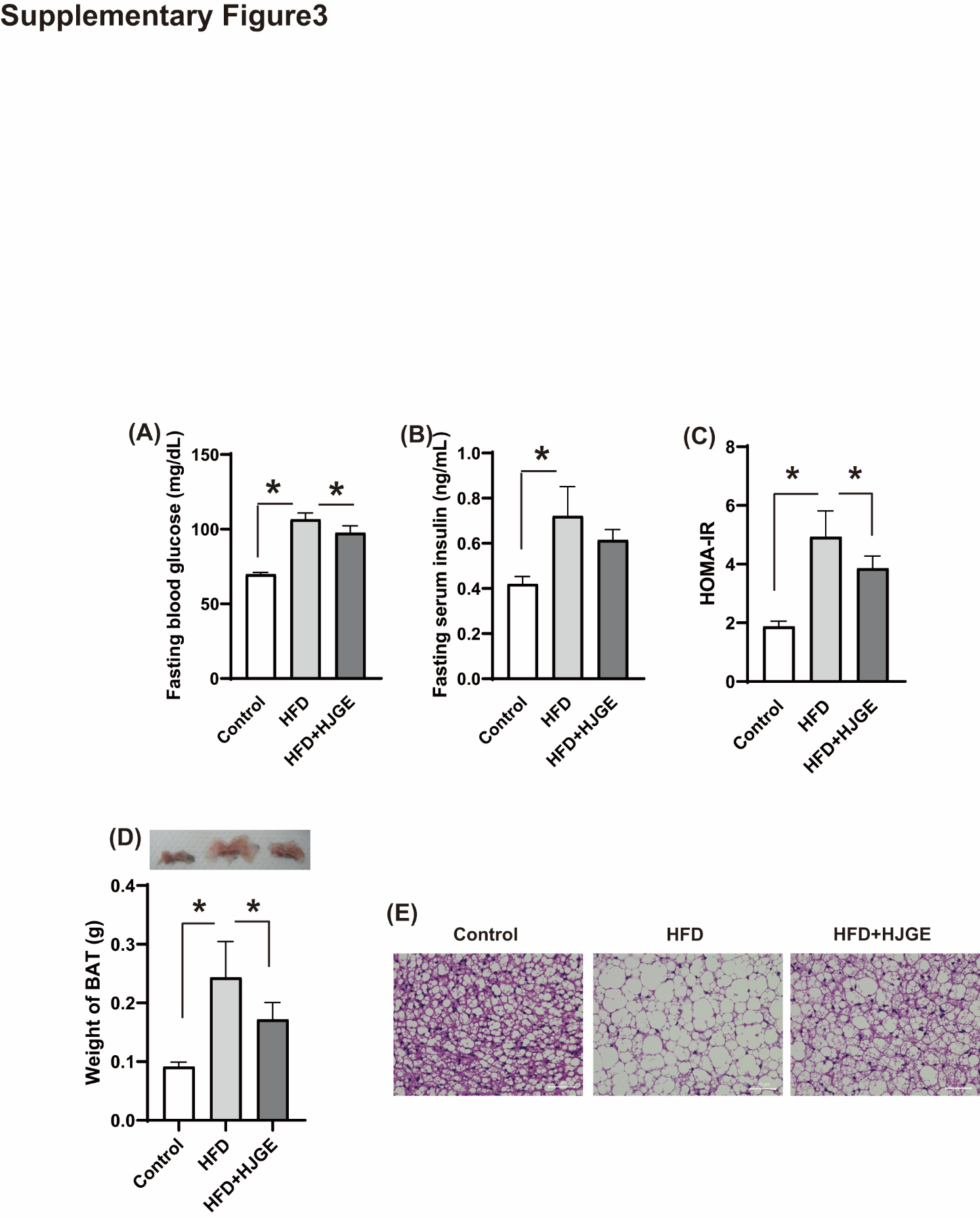


**Supplementary** **Figure 3.** **Effects of HJGE on glucose metabolism and morphological change of BAT in HFD-induced obesity**

Six-week-old male *C57BL/6J* mice were fed an HFD for four weeks and then divided into three diet groups fed a chow diet (control), HFD, and HFD supplemented with 3.8% HJGE (HFD+HJGE). (A) Fasting blood glucose, (B) fasting insulin level, and (C) HOMA-IR (n = 5-6). (D) Representative photograph of BAT of mice fed the indicated diet (upper) and weight of BAT following the feeding of the indicated diets (lower) (n = 5-7). (E) Representative hematoxylin–eosin-staining images of BAT; scale bar, 50 μm. Data are shown as mean ± SD. One-way ANOVA followed by Tukey’s post hoc test. **P* < 0.05. HOMA-IR, homeostasis model assessment as an index of insulin resistance.

**
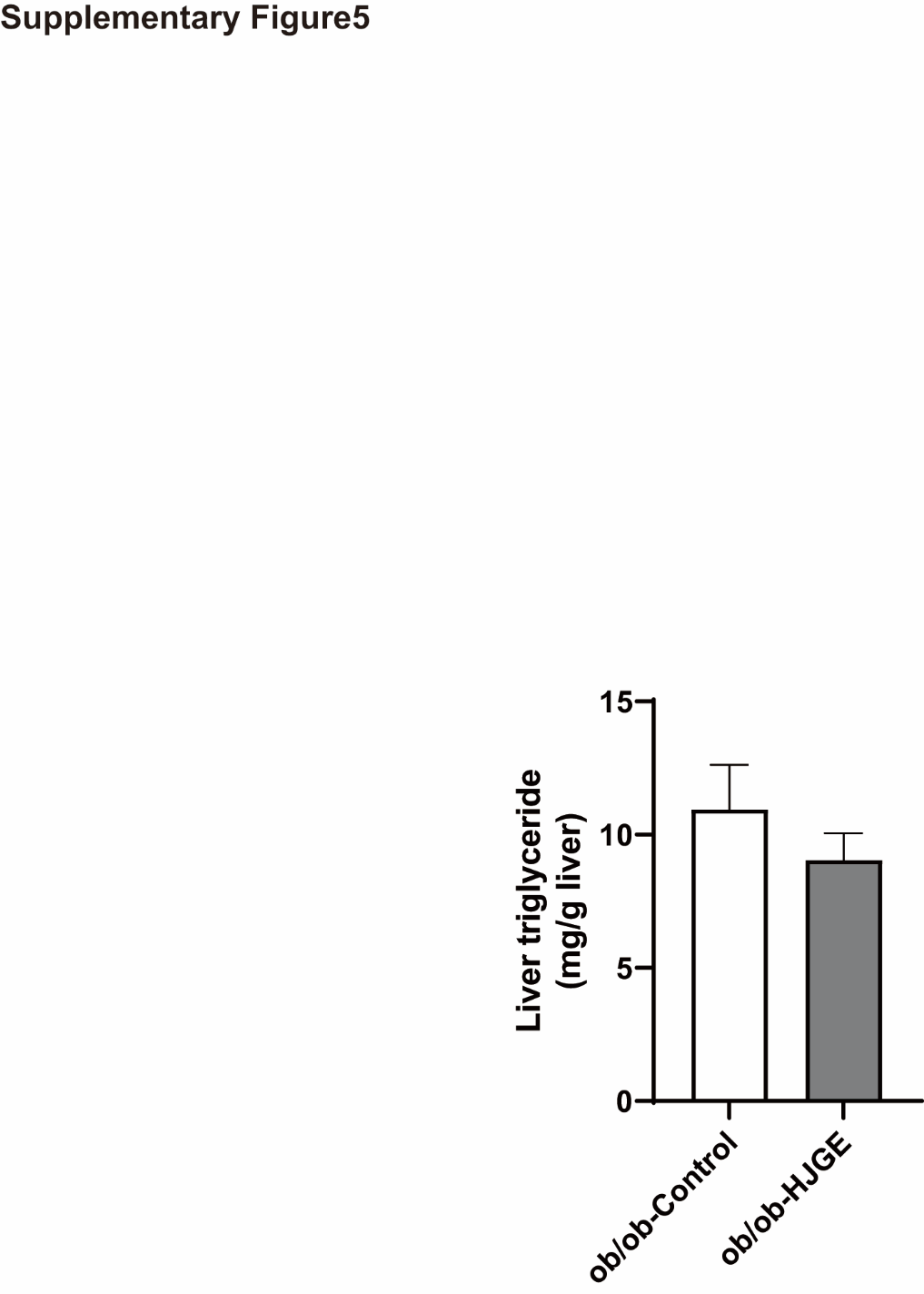
**

**Supplementary Figure 4. Effects of HJGE on hepatic triglyceride accumulation in the livers of *ob/ob* mice.**

Triglyceride levels in the liver (n = 3). Data are shown as mean ± SD. Two-tailed unpaired Student's *t-*test.


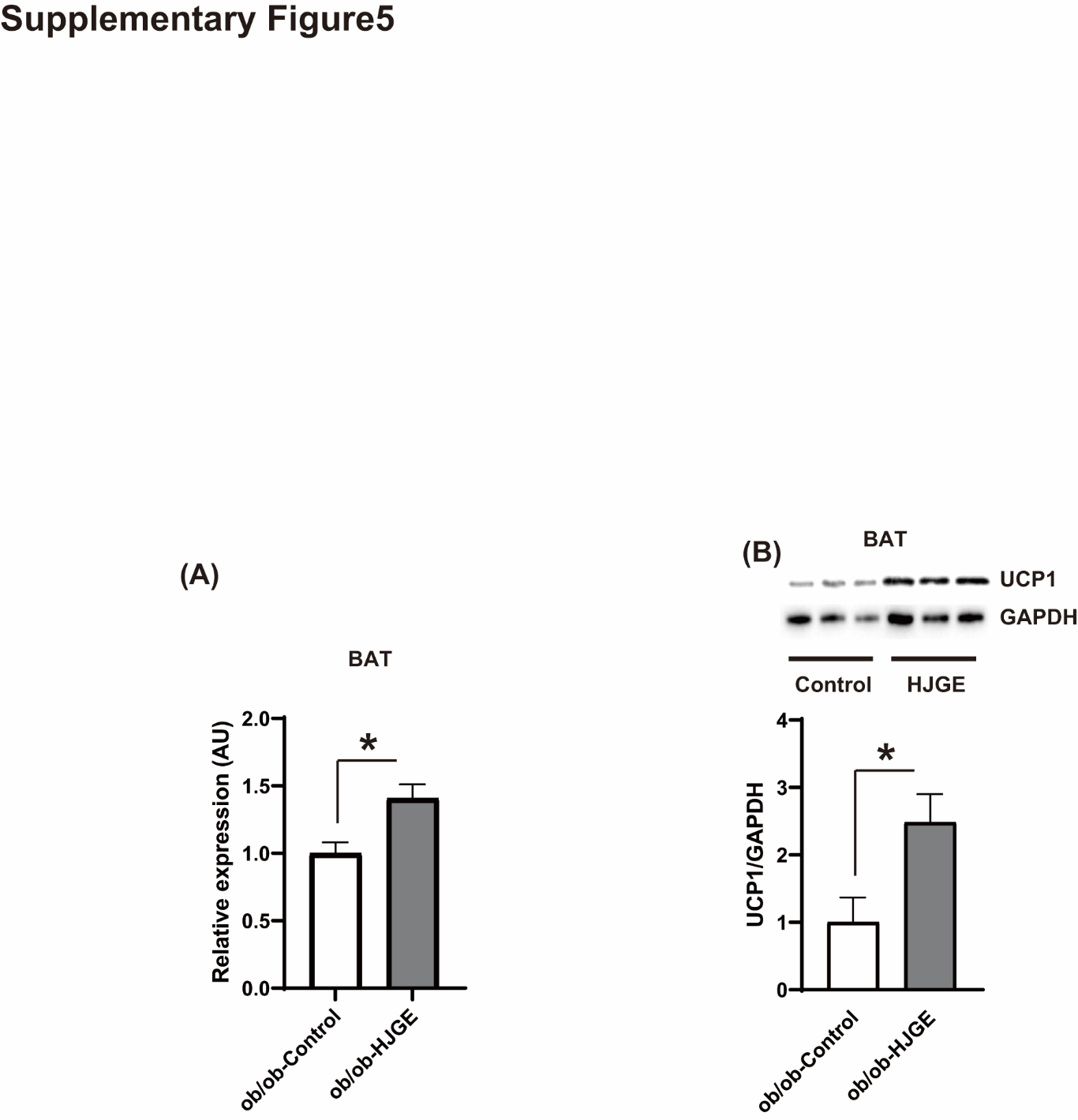


**Supplementary Figure 5.** **Effects of HJGE on Ucp1 gene and protein expressions in BAT of *ob/ob* mice.**

(A) mRNA levels of the *Ucp1* gene (n = 3) and (B) immunoblot for UCP1 and quantification of the protein expression normalized by GAPDH (n = 3) in BAT of HJGE-treated *ob/ob* mice. Relative gene and protein expression is shown as mean ± SD. Significance was evaluated using the two-tailed unpaired Student's *t-*test. **P* < 0.05.

## Supplementary Tables

**Supplementary Table 1**. Crude drugs in HJGE and their plants of origin.

**
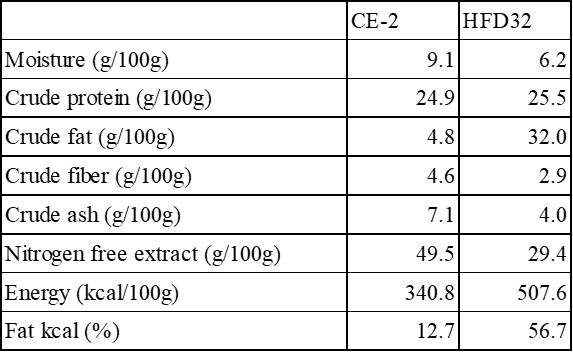
Supplementary Table 2.** Main ingredients of the chow diet and high-fat diet.

**Supplementary Table 3**. Primers used in real-time PCR.

**Supplementary Table 4**. Antibodies used in immunoblotting.

Primary antibodies

Secondary antibodies
